# Prognostic Role of TERT Mutations in Chondrosarcoma: Associations with Dedifferentiation, Survival, and IDH/TERT Co-Mutations

**DOI:** 10.3390/cancers18142272

**Published:** 2026-07-15

**Authors:** Alyan Zafar, Daisy Ference, Brooke M. Crawford, Sergio Jose Torralbas Fitz, Francis J. Hornicek, H. Thomas Temple

**Affiliations:** Department of Orthopedics, Musculoskeletal Oncology Division, University of Miami Heath System, Miami, FL 33136, USA; axz491@med.miami.edu (A.Z.); daf199@miami.edu (D.F.); bxc859@med.miami.edu (B.M.C.); sjt96@med.miami.edu (S.J.T.F.); fjh21@med.miami.edu (F.J.H.)

**Keywords:** chondrosarcoma, *TERT* promoter mutation, *IDH* mutation, next-generation sequencing, overall survival, dedifferentiated chondrosarcoma, prognostic biomarkers

## Abstract

**Background/Objectives**: Chondrosarcoma is a clinically heterogeneous malignancy, and the prognostic role of its genetic features remains incompletely defined. While isocitrate dehydrogenase (*IDH*) mutations are well characterized, the significance of less common alterations—particularly telomerase reverse transcriptase (*TERT*) promoter mutations—remains unclear. This study aimed to define the genomic profile of chondrosarcoma and evaluate the prognostic relevance of *TERT* mutations, alone and in combination with *IDH* mutations. **Methods**: We retrospectively analyzed 91 patients with chondrosarcoma, including 54 patients with available next-generation sequencing data for genomic analyses. Tumors were assessed for mutations in *IDH, TERT, TP53,* and groups of genes involved in key cellular functions (e.g., cell cycle control, chromatin regulation). Associations with tumor characteristics were evaluated, recurrence outcomes were assessed using logistic regression, and overall survival was analyzed using Kaplan–Meier and Cox models adjusted for tumor subtype, grade, and size. **Results**: *IDH* mutations were present in 52% of tumors; other alterations included TP53 (16%), *TERT* (7.7%), chromatin-related genes (14%), and cell cycle genes (8.8%). *TERT* mutations were enriched in dedifferentiated tumors (*p* = 0.017) and occurred exclusively in grade 3 disease (“*p* < 0.001”). *TERT*-mutant tumors were associated with worse overall survival upon unadjusted analysis (HR 7.02, *p* = 0.024), but not after adjustment (HR 3.81, *p* = 0.160). All *TERT* mutations co-occurred with *IDH* mutations, and this subgroup had particularly poor survival (HR 7.76, *p* = 0.022), albeit limited by small numbers. **Conclusions**: These findings suggest that specific mutations, alone or in combination, may influence clinical outcomes by reflecting more aggressive tumor biology. *TERT* mutations, particularly with concurrent *IDH* mutations, may identify high-risk patients and complement established prognostic factors. Further validation is needed.

## Simple Summary

Chondrosarcoma is a rare bone cancer that can range from slow-growing tumors to highly aggressive cancers with poor outcomes. Identifying genetic changes associated with aggressive disease may help improve risk stratification and patient management. In this study, we analyzed genetic sequencing data from 54 patients within a cohort of 91 patients with chondrosarcoma to determine whether mutations in the *TERT* gene were associated with aggressive tumor behavior and survival. We found that *TERT* mutations were strongly linked to dedifferentiated and high-grade tumors, both of which are known to be associated with worse clinical outcomes. Patients with *TERT*-mutated tumors had poorer survival, although this relationship was largely explained by the aggressive characteristics of the tumors themselves. Importantly, patients harboring both *IDH* and *TERT* mutations experienced the worst survival outcomes, although these findings were based on a small number of cases and require further validation. These findings suggest that *TERT* mutations may serve as a marker of aggressive tumor biology and that combined *IDH/TERT* mutation status may help identify particularly high-risk patients.

## 1. Introduction

Chondrosarcoma is the second-most common primary malignant bone tumor in adults and is defined by the production of cartilaginous matrix and a broad spectrum of clinical behavior. Tumor behavior ranges from indolent tumors with low recurrence risk to aggressive disease with metastasis and disease-specific mortality. Surgical resection is the mainstay of treatment, as conventional chondrosarcomas respond poorly to chemotherapy and radiotherapy [1]. Accurate prognostic stratification is therefore essential to guide surgical planning, surveillance, and patient counseling [2,3].

Current prognostic assessment relies predominantly on clinicopathologic variables, including histologic grade, tumor subtype, and disease stage. Among these, histologic grade is the most important predictor of outcome, with higher-grade tumors being associated with increased rates of recurrence and death. Dedifferentiated chondrosarcoma represents a highly aggressive subtype characterized by rapid progression and poor survival, whereas clear cell chondrosarcoma generally demonstrates a more favorable clinical course [4,5]. Despite their clinical utility, these parameters do not fully account for the biologic heterogeneity observed in practice, where tumors with similar histologic features may exhibit markedly different clinical behaviors. These limitations underscore the need for additional prognostic markers that more accurately reflect tumor biology [6,7].

Advances in next-generation screening (NGS) have led to better understanding of the molecular landscape of chondrosarcoma. Mutations in *IDH1* and *IDH2* are among the most frequently identified alterations, present in approximately half of cases, and are believed to represent early events in tumorigenesis [8,9,10,11]. Additional genomic alterations have been described, including mutations in *TP53*, cell cycle regulatory genes such as *CDKN2A* and *RB1*, and genes involved in chromatin remodeling, including *ATRX* and members of the *KMT2* family [12,13]. These findings have expanded the molecular characterization of chondrosarcoma; however, the clinical relevance of many of these alterations remains uncertain. Notably, although *IDH* mutations are common, their association with survival outcomes has been inconsistent, suggesting that frequently occurring mutations do not necessarily confer prognostic significance [14].

*TERT* promoter mutations represent a distinct class of genomic alterations that may have greater relevance to tumor progression. By promoting telomerase activation, *TERT* mutations enable cellular immortalization and have been associated with aggressive tumor behavior and adverse outcomes across a range of malignancies [15,16,17,18]. In chondrosarcoma, emerging evidence suggests that *TERT* promoter mutations are associated with higher-grade tumors and disease progression, suggesting a role in later stages of tumor evolution [19]. Through telomerase activation and maintenance of replicative capacity, *TERT* alterations may facilitate escape from senescence and contribute to progression toward dedifferentiated and metastatic disease states [20]. As these alterations are thought to occur later in tumor evolution—unlike early driver mutations such as *IDH*—this supports a stepwise model in which initial *IDH*-driven tumorigenesis is followed by additional changes that promote aggressive transformation [21,22]. Despite an increasing focus on *TERT* promoter alterations, their precise impact on chondrosarcoma survival and disease progression remains nuanced and uncertain. It is unclear whether *TERT* mutations independently predict survival or primarily reflect established high-risk clinicopathologic features such as grade and dedifferentiation. Furthermore, the relationship between *TERT* mutations and other genomic alterations, particularly *IDH* mutations, has not been fully elucidated, limiting understanding of how these alterations may interact within the broader molecular framework of the disease. Although prior studies have demonstrated associations between *TERT* promoter mutations and adverse outcomes in chondrosarcoma, many have focused primarily on mutation prevalence or unadjusted survival associations and have not evaluated *TERT* within the context of other genomic alterations and established clinicopathologic predictors. Furthermore, the prognostic implications of concurrent *IDH* and *TERT* mutations remain poorly characterized. Accordingly, the present study sought to integrate molecular profiling with traditional prognostic variables to better define the biologic and clinical significance of *TERT* alterations in chondrosarcoma.

The purpose of this study was to evaluate the association between *TERT* mutations and clinicopathologic features, including tumor grade and histologic subtype, and to assess their relationship with overall survival in a cohort of patients with chondrosarcoma. We further sought to determine whether *TERT* mutations provide independent prognostic information beyond established clinical factors and to explore their interaction with *IDH* mutations. We hypothesized that *TERT* mutations would be associated with aggressive tumor characteristics and worse survival, and that it may identify a high-risk subset within *IDH*-mutant chondrosarcomas.

## 2. Materials and Methods

### 2.1. Study Design and Patient Selection

This study was conducted as a retrospective cohort analysis at the University of Miami Health System. Patients with a histopathological diagnosis of chondrosarcoma treated at our institution were identified from a comprehensive institutional database. Patients were included if they had a histopathological diagnosis of chondrosarcoma and available clinical data. Among these patients, 54 had available next-generation sequencing data and were included in genomic analyses, whereas patients without genomic testing were treated as missing for marker-specific analyses. Genomic survival analyses were therefore restricted to the subset of patients with available genomic testing data. Patients with missing key clinical or survival data were excluded from survival analyses.

### 2.2. Data Collection

Clinical, pathologic, and genomic data were extracted from the institutional electronic medical record and added to a comprehensive dataset that included the following variables: age, sex, date of surgery, and last follow-up or date of death.

Tumor characteristics included histological subtype (conventional, dedifferentiated, or clear cell), tumor grade (grade 2 vs. grade 3), tumor size, tumor stage at presentation, and tumor location (Axial vs. Appendicular). Recurrence and metastatic status were also recorded.

**Figure 1:**
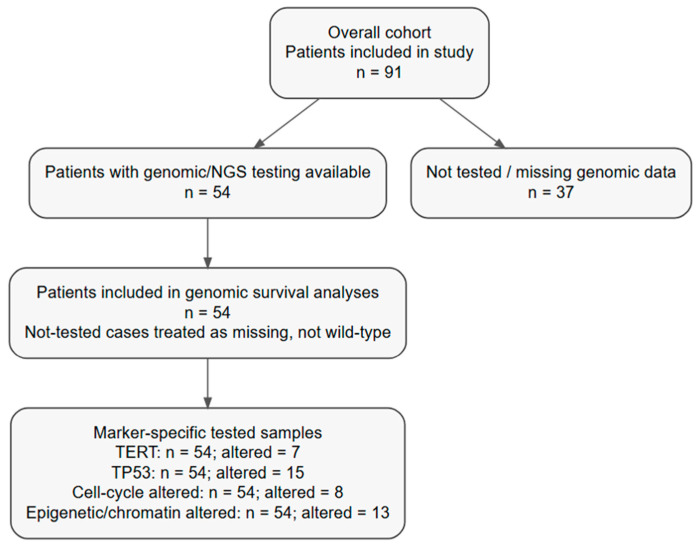
Study flow diagram demonstrating patient inclusion and availability of genomic testing for survival analyses.

Genomic data was obtained from NGS reports and included mutation status for *IDH*, *TP53*, *TERT*, cell cycle regulatory genes (*CDKN2A*, *CDKN2B*, *RB1*), and chromatin remodeling genes (e.g., *ATRX*; *KMT2C*; *KMT2D*; *KMT2A*; *SETD2*; *BCOR*; *EED*). Mutation variables were categorized as present, absent, or not tested. Genomic marker analyses were performed among patients with available testing for each marker. For the four key markers evaluated in survival analyses (*TERT*, *TP53*, cell cycle regulatory genes, and chromatin remodeling genes), testing was available in 54 of 91 patients, while 37 patients lacked genomic testing and were treated as missing (Figure 1). Consequently, genomic survival analyses were restricted to the tested subset, reducing sample size and statistical power. Because genomic testing was more likely to be performed in patients with clinically aggressive disease or consideration for targeted therapies, missingness may have introduced selection bias and influenced effect estimates.

### 2.3. Outcome

The primary outcome was overall survival, defined as the time from the date of diagnosis to death or last follow-up. Patients who were alive at last follow-up were censored. Secondary outcomes include chondrosarcoma recurrence.

### 2.4. Statistics

All statistical analyses were performed using R Core Team (Version 4.4.1). Continuous variables are presented as mean ± standard deviation, and categorical variables are reported as frequencies and percentages. Due to the limited number of clear cell chondrosarcomas (n = 3), inferential comparisons of patient clinicopathologic characteristics were restricted to conventional and dedifferentiated chondrosarcoma. Continuous variables were compared using the Wilcoxon rank-sum test, while categorical variables were compared using Fisher’s exact test.

Associations between TERT mutations, histological subtype, and grade were assessed using chi-square tests or Fisher’s exact tests when appropriate. Logistic regression analyses were additionally performed to evaluate associations between genomic alterations and recurrence outcomes. Odds ratios (ORs) with 95% confidence intervals were calculated for recurrence following surgery. Similar analyses were performed for other genomic alterations, including *IDH* mutations, *TP53* mutations, cell cycle regulatory gene alterations, and epigenetic/chromatin remodeling pathway alterations. To characterize differences in clinical outcomes between tumor subtypes, logistic regression analyses were performed to evaluate associations between histological subtype and recurrence after surgery as well as stage progression during follow-up. Conventional chondrosarcoma served as the reference category, and odds ratios with 95% confidence intervals were reported.

Overall survival was defined as the time from date of diagnosis to death or last follow-up. Survival distributions were estimated using the Kaplan–Meier method and compared using the log-rank test. To evaluate associations between variables and overall survival, a Cox proportional hazards regression model was used. Univariable analysis was performed for *TERT* mutations, tumor grade, histological subtype, tumor size, and other genomic alterations. Multivariable Cox regression models were used to assess the independent effect of *TERT* mutations after adjusting for histological subtype, tumor size, and tumor grade. Lastly, a Cox regression was performed to evaluate the combined effect of *IDH* and *TERT* mutation on overall survival by stratifying patients into four groups: *IDH* wild-type/*TERT* wild-type, *IDH*-mutant/*TERT* wild-type, *IDH* wild-type/*TERT*-mutant, and *IDH*-mutant/*TERT*-mutant. Hazard ratios are reported with 95% confidence intervals (CI). The proportional hazards assumption for Cox regression models was assessed using Schoenfeld residual testing. No significant violations of proportional hazards were observed for *TERT*, *TP53*, cell-cycle alterations, chromatin remodeling alterations, or IDH subtype analyses (all *p* > 0.05).

All tests were two-sided, and a *p*-value < 0.05 was considered statistically significant.

## 3. Results

### 3.1. Clinical Data and Patient Demographics

A total of 91 patients with chondrosarcoma were included in the study cohort, of whom 54 had available next-generation sequencing data for genomic analyses. Overall, the study cohort consisted of 59 conventional, 29 dedifferentiated, and 3 clear cell chondrosarcomas. Among them, 56% of patients were male and 44% were female. The majority of patients were White (87%) and non-Hispanic (64%). Mean body mass index (BMI) for the overall cohort was 27.75 ± 5.67 kg/m^2^. Most tumors were located in the axial skeleton (62%), while 38% arose in the appendicular skeleton. Compared with conventional chondrosarcoma, dedifferentiated tumors demonstrated significantly higher rates of grade 3 disease (76% vs. 14%, *p* < 0.001). Dedifferentiated tumors also exhibited larger mean tumor size (8.92 ± 3.46 cm vs. 7.41 ± 3.93 cm, *p* = 0.083), more advanced disease at final follow-up, with 76% classified as stage IV compared with 41% of conventional tumors (stage progression analysis, *p* = 0.200), and higher rates of distant metastatic recurrence (52% vs. 36%, *p* = 0.105), although these differences did not reach statistical significance. Positive surgical margins were identified in 13% of tumors overall and were not significantly different between tumor subtypes (*p* = 0.492). Recurrence after initial surgery occurred in 55% of patients overall and was more common among dedifferentiated tumors (59%) than conventional tumors (54%), demonstrating a trend toward increased recurrence risk that did not reach statistical significance (OR 2.76, 95% CI 0.95–9.32; *p* = 0.076). At final follow-up, 31% of patients had died, including 30% who died of disease. Mortality was substantially higher among patients with dedifferentiated chondrosarcoma, with 45% deceased at final follow-up compared with 25% of patients with conventional tumors. Overall survival was significantly worse among patients with dedifferentiated chondrosarcoma (HR 2.91, 95% CI 1.37–6.18; *p* = 0.005) (Table 1).

### 3.2. Genomic Landscape of Chondrosarcoma Mutations

Among patients who underwent NGS, genomic alterations were identified across multiple pathways. *IDH* mutations were the most common alteration, present in 52% of tumors. *TERT* promoter mutations were identified in 7.7% of patients, while *TP53* mutations were observed in 16% of cases. Additional alterations were observed in cell cycle regulatory genes (*CDKN2A, CDKN2B, RB1*) as well as in chromatin remodeling genes (e.g., *ATRX; KMT2C; KMT2D; KMT2A; SETD2; BCOR; EED*). NGS showed that 8.8% of cases exhibited mutations in cell cycle regulatory genes and 14% of cases exhibited mutations in chromatin remodeling genes (Table 2). Overall, these findings demonstrate a heterogeneous genomic landscape that is consistent with the literature.

### 3.3. TERT Mutations Are Associated with Aggressive Tumor Biology and Adverse Clinical Outcomes

**Figure 2:**
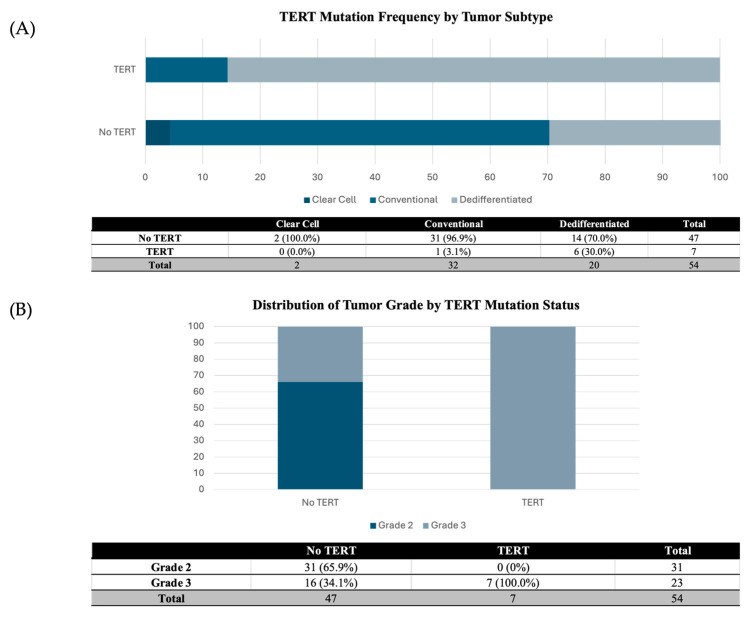
Association of *TERT* promoter mutations with histologic subtype and tumor grade in chondrosarcoma. (**A**) *TERT* mutations were significantly enriched in dedifferentiated chondrosarcoma compared with conventional and clear cell subtypes (*p* = 0.017). (**B**) *TERT* mutations were exclusively observed in grade 3 tumors and were significantly associated with high-grade disease (*p* < 0.001).

*TERT* promoter mutations demonstrated a differential distribution across histological subtypes and were significantly enriched in dedifferentiated chondrosarcoma (*p* = 0.017). *TERT* mutations were identified in 30.0% of dedifferentiated tumors, compared to 3.1% of conventional tumors and 0% of clear cell tumors. Notably, 85.7% of all *TERT*-mutated tumors occurred in dedifferentiated tumors (Figure 2A).

Additionally, *TERT* promoter mutations were significantly associated with higher tumor grade (“*p* < 0.001”). All *TERT*-mutated tumors (100%) were grade 3 chondrosarcomas, whereas no *TERT* mutations (0.0%) were identified in grade 2 chondrosarcomas. Among *TERT* wild-type tumors, 65.9% were grade 2 chondrosarcomas and 34.1% were grade 3 chondrosarcomas (Figure 2B). Together, these findings demonstrate that *TERT* promoter mutations are strongly associated with both dedifferentiated histology and high-grade chondrosarcoma, supporting their role as a marker of aggressive tumor biology.

**Figure 3:**
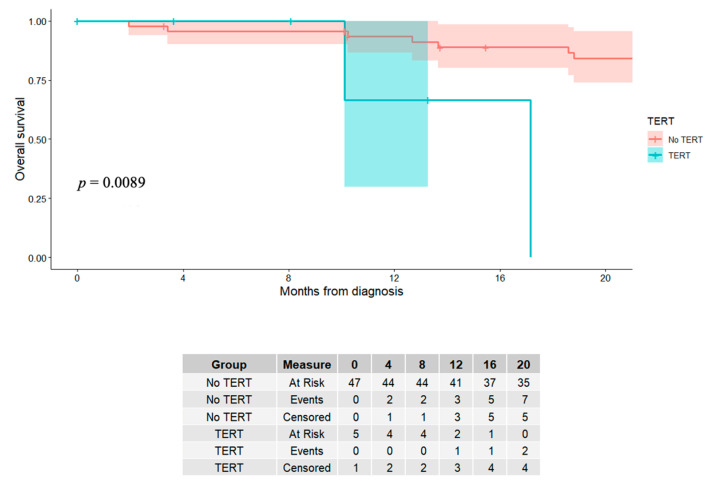
Kaplan–Meier overall survival analysis according to *TERT* mutation status. *TERT*-mutant tumors demonstrated significantly worse overall survival compared with *TERT* wild-type tumors (HR 7.02, 95% CI 1.30–38.02; *p* = 0.024; log-rank *p* = 0.0089). During follow-up, 7 of 47 patients with *TERT* wild-type tumors and 2 of 5 patients with *TERT*-mutant tumors experienced the event of interest (death).

**Figure A1:**
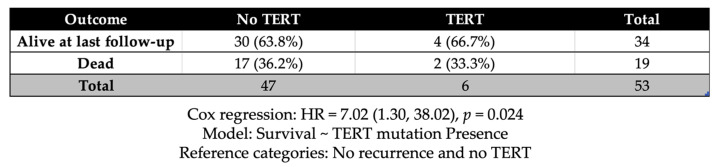
Univariable Cox regression demonstrating worse overall survival among patients with TERT-mutated chondrosarcoma (HR 7.02, 95% CI 1.30–38.02; *p* = 0.024).

**Figure A2:**
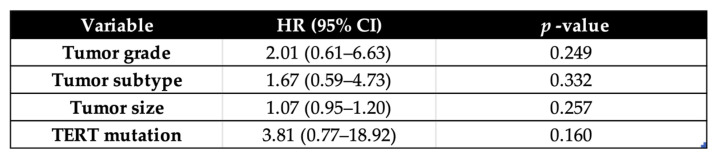
Multivariable Cox regression model for overall survival. Hazard ratios, 95% confidence intervals (CI), and *p*-values are reported for each comparison.

Given *TERT’s* role as a marker of aggressive tumor biology, survival analysis was performed. In exploratory survival analysis, *TERT* promoter mutations were significantly associated with decreased overall survival. Upon Kaplan–Meier analysis, patients with *TERT*-mutant tumors had worse overall survival than those without (log-rank *p* = 0.0089). During follow-up, death occurred in 2 of 5 (40%) patients with *TERT*-mutant tumors compared with 7 of 47 (14.9%) patients with *TERT*-wild-type tumors (Figure 3). Additionally, in univariable Cox regression, *TERT* mutations were associated with increased mortality risk (HR 7.02, 95% CI 1.30–38.02; *p* = 0.024) (Figure A1). This association was not retained after adjustment for tumor grade, size, and subtype (HR 3.81, 95% CI 0.77–18.92; *p* = 0.160), suggesting *TERT* may reflect tumor aggressiveness rather than act as an independent prognostic factor (Figure A2).

**Figure A3:**
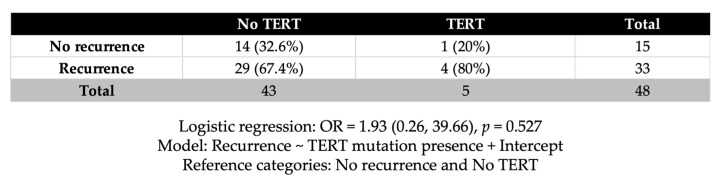
Logistic regression analysis of recurrence according to TERT mutation status.

In recurrence analysis, *TERT*-mutated tumors showed a higher recurrence proportion compared with *TERT* wild-type tumors, although this association was not statistically significant (OR = 1.93, 95% CI 0.26–39.66; *p* = 0.527). This suggests a possible trend toward greater recurrence among *TERT*-mutated tumors, but the small number of *TERT*-positive cases limits statistical interpretation (Figure A3).

### 3.4. Other Genomic Alterations and Survival

**Figure 4:**
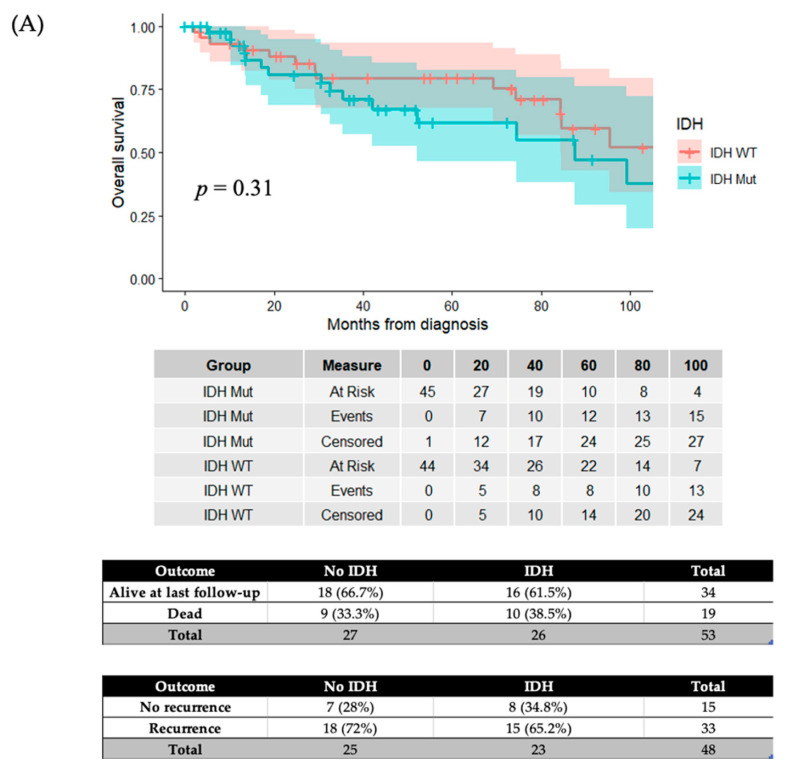

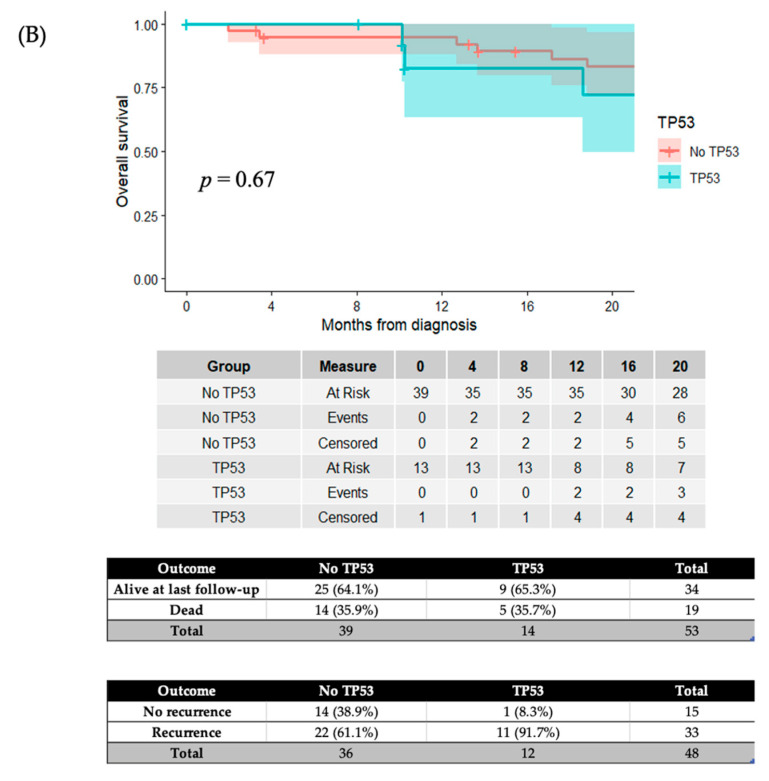
Association of *IDH* and *TP53* mutations with overall survival and recurrence in chondrosarcoma. (**A**) *IDH* mutation status was not significantly associated with overall survival (HR 1.47, 95% CI 0.56–3.62; *p* = 0.408; log-rank *p* = 0.31) or recurrence (OR 0.73, 95% CI 0.21–2.49; *p* = 0.613). During follow-up, 13 of 44 patients with *IDH*-wild-type tumors and 15 of 45 patients with *IDH*-mutant tumors experienced death. (**B**) *TP53* mutations were not associated with overall survival (HR 1.25, 95% CI 0.45–3.45; *p* = 0.670; log-rank *p* = 0.67) but demonstrated a trend toward increased recurrence risk that did not reach statistical significance (OR 7.00, 95% CI 1.16–135.12; *p* = 0.077). During follow-up, 6 of 39 patients without *TP53* mutations and 3 of 13 patients with *TP53* mutations experienced death.

**Figure A4:**
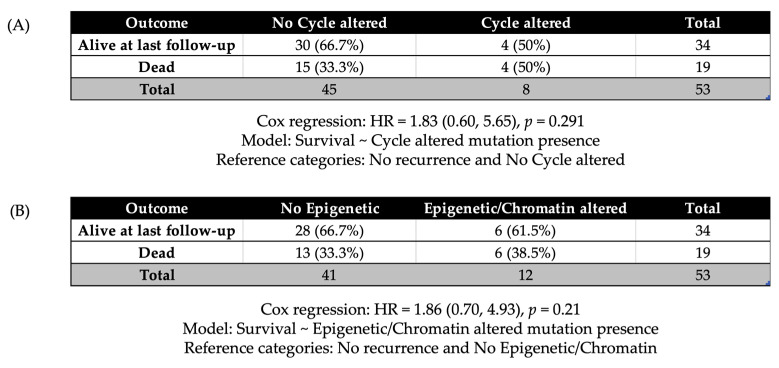
Cox regression analyses evaluating survival according to (**A**) cell cycle regulatory gene alterations (HR 1.83, 95% CI 0.60–5.65; *p* = 0.291) and (**B**) epigenetic/chromatin remodeling gene alterations (HR 1.86, 95% CI 0.70–4.93; *p* = 0.210).

Additional genomic alterations were evaluated for their association with overall survival including *IDH, TP53*, cell cycle regulatory genes, and chromatin remodeling pathways. *IDH* mutations were not significantly associated with overall survival on Cox regression (HR= 1.47, 95% CI 0.56–3.62; *p* = 0.408) and KM analysis (log-rank *p* = 0.31). Death occurred in 15 of 45 (33.3%) patients with *IDH*-mutant tumors and 13 of 44 (29.5%) patients with *IDH*-wild-type tumors (Figure 4A). Similarly, *TP53* mutations were not associated with survival outcomes on Cox regression (HR = 1.25, 95% CI 0.45–3.45; *p* = 0.670) and KM analysis (log-rank *p* = 0.67). During follow-up, death occurred in 3 of 13 (23.1%) patients with TP53-mutant tumors compared with 6 of 39 (15.4%) patients with *TP53*-wild-type tumors (Figure 4B). A similar trend followed for cell cycle regulatory genes (HR 1.83, 95% CI 0.60–5.65; *p* = 0.291) and chromatin remodeling pathways (HR 1.86, 95% CI 0.70–4.93; *p* = 0.210) (Figure A4).

**Figure A5:**
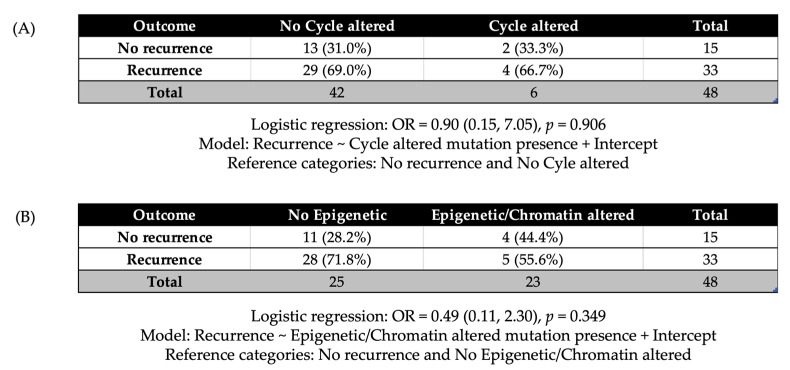
Logistic regression analyses evaluating recurrence according to (**A**) cell cycle regulatory gene alterations (OR 0.90, 95% CI 0.15–7.05; *p* = 0.906) and (**B**) epigenetic/chromatin remodeling gene alterations (OR 0.49, 95% CI 0.11–2.30; *p* = 0.349).

Recurrence analyses demonstrated no significant association between IDH mutation status and recurrence after surgery (OR = 0.73, 95% CI 0.21–2.49; *p* = 0.613) (Figure 4A). In contrast, *TP53* mutations demonstrated a strong trend toward increased recurrence risk (OR = 7.0, 95% CI 1.16–135.12; *p* = 0.077), although this did not reach statistical significance (Figure 4B). Alterations involving cell cycle regulatory genes (OR 0.90, 95% CI 0.15–7.05; *p* = 0.906) and chromatin remodeling pathways (OR 0.49, 95% CI 0.11–2.30; *p* = 0.349) were likewise not significantly associated with recurrence outcomes (Figure A5).

**Figure A6:**
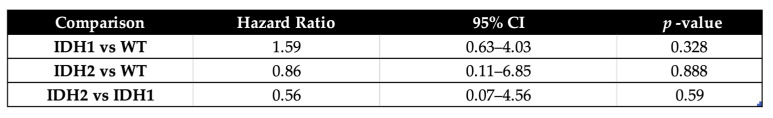
Sensitivity analysis of overall survival according to IDH mutation subtype using univariable Cox proportional hazards regression. Hazard ratios, 95% confidence intervals (CI), and *p*-values are reported for each comparison.

In an exploratory sensitivity analysis, *IDH1*- and *IDH2*-mutant tumors were evaluated separately. Compared with *IDH*-wild-type tumors, *IDH1*-mutant tumors demonstrated an HR of 1.59 (95% CI 0.63–4.03; *p* = 0.328), whereas *IDH2*-mutant tumors demonstrated an HR of 0.86 (95% CI 0.11–6.85; *p* = 0.888). Direct comparison of *IDH2*- versus *IDH1*-mutant tumors similarly demonstrated no statistically significant difference in overall survival (HR 0.56, 95% CI 0.07–4.56; *p* = 0.590) (Figure A6).

Overall, aside from *TERT* alterations, no other genomic changes demonstrated a significant association with survival outcomes within this cohort. However, the observed recurrence trend among *TP53*-mutated tumors may suggest a potential role in aggressive tumor behavior that warrants further investigation in larger studies.

### 3.5. Established Clinicopathological Predictors of Survival

**Figure 5:**
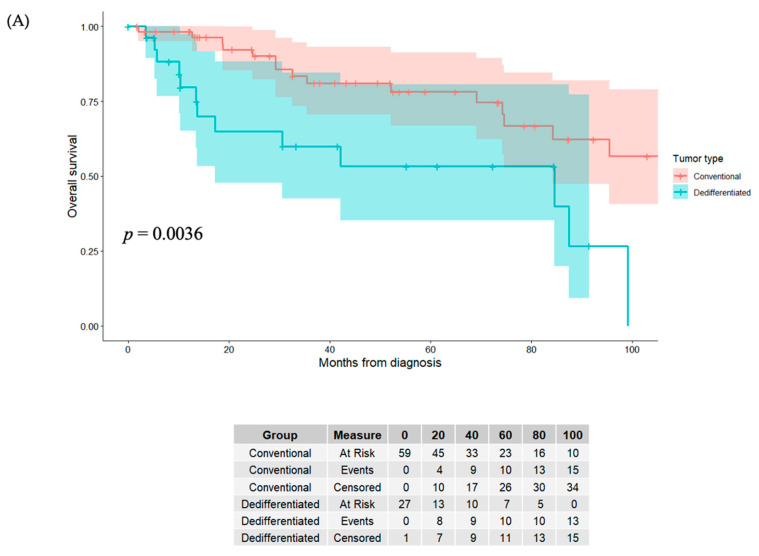

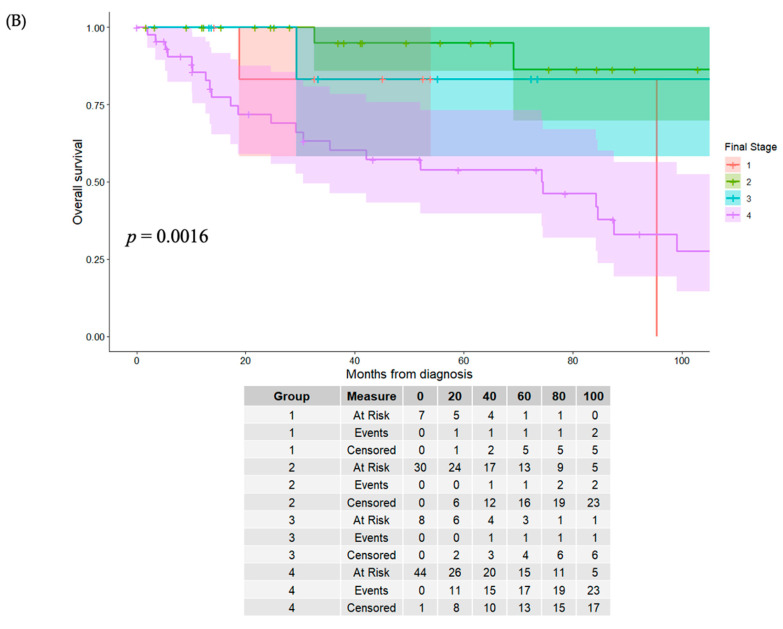

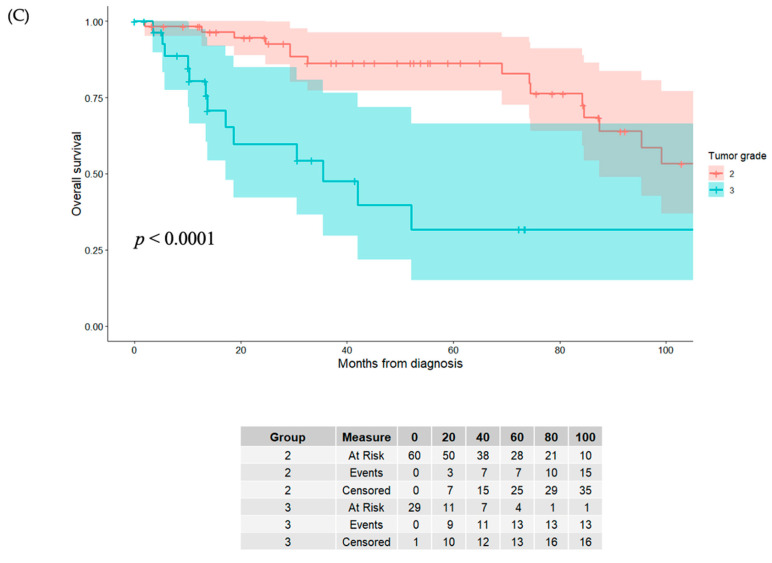
Overall survival stratified by histologic subtype, final stage, and tumor grade. (**A**) Dedifferentiated histology was associated with worse overall survival for Cox regression (HR 2.91, 95% CI 1.37–6.18; *p* = 0.005) and Kaplan–Meier analysis (log-rank *p* = 0.0036). During follow-up, 15 of 59 patients with conventional chondrosarcoma and 13 of 27 patients with dedifferentiated chondrosarcoma died. (**B**) Advanced stage was associated with significantly worse overall survival (log-rank *p* = 0.0016). During follow-up, deaths occurred in 2 of 7 stage I patients, 2 of 30 stage II patients, 1 of 8 stage III patients, and 23 of 44 stage IV patients. (**C**) Grade 3 disease was associated with significantly worse survival (“log-rank *p* < 0.0001”), with 13 of 29 grade 3 patients and 15 of 60 grade 2 patients experiencing death during follow-up.

**Figure A7:**
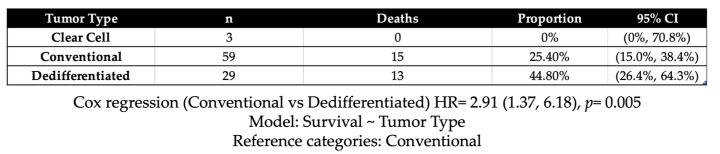
Cox regression analysis demonstrating worse survival in dedifferentiated versus conventional chondrosarcoma (HR 2.91, 95% CI 1.37–6.18; *p* = 0.005).

Established clinicopathological variables such as dedifferentiated histology, stage progression, and tumor grade were significantly associated with overall survival. Dedifferentiated histology was associated with worse survival compared to conventional tumors in both Cox regression (HR 2.91, 95% CI 1.37–6.18; *p* = 0.005) (Figure A7) and KM analysis (log-rank *p* = 0.0036), with death occurring in 13 of 27 (48.1%) patients with dedifferentiated chondrosarcoma compared with 15 of 59 (25.4%) patients with conventional chondrosarcoma (Figure 5A). Patients with higher final stage also demonstrated significantly worse overall survival upon Kaplan–Meier analysis (log-rank *p* = 0.0016), with deaths occurring in 2 of 7 (28.6%) stage I patients, 2 of 30 (6.7%) stage II patients, 1 of 8 (12.5%) stage III patients, and 23 of 44 (52.3%) stage IV patients. (Figure 5B).

Higher tumor grade was significantly associated with decreased overall survival. Patients with grade 3 tumors demonstrated worse survival on KM analysis compared to those with grade 2 tumors (log-rank “*p* <0.0001”), with death occurring in 13 of 29 (44.8%) grade 3 patients compared with 15 of 60 (25.0%) grade 2 patients (Figure 5C). These findings reflect the more aggressive biology of higher tumor grade. Overall, these findings are consistent with prior literature and support the internal validity of the cohort.

### 3.6. IDH-TERT Co-Mutation Analysis

**Figure 6:**
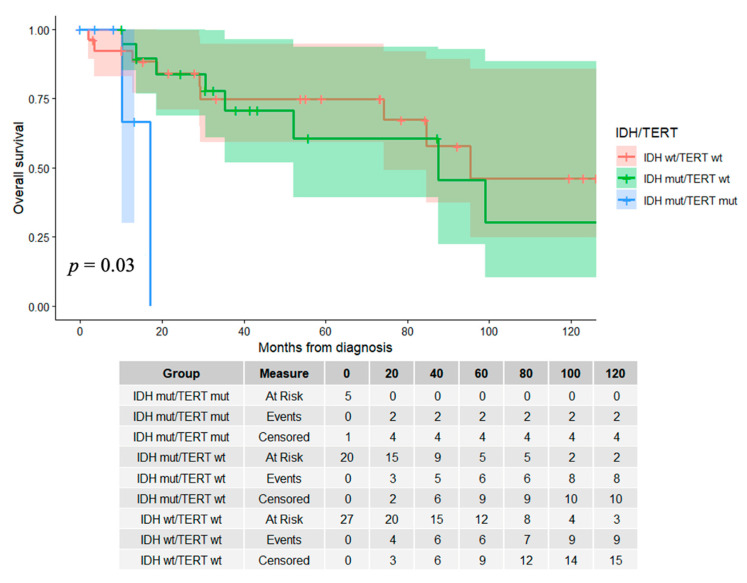
Kaplan–Meier overall survival analysis stratified by combined *IDH* and *TERT* mutation status. Concurrent *IDH/TERT* mutations identified a high-risk subgroup with significantly worse survival (HR 7.76, 95% CI 1.34–44.85; *p* = 0.022; log-rank *p* = 0.03). During follow-up, death occurred in 9 of 27 patients with *IDH*-wild-type/*TERT*-wild-type tumors, 8 of 20 patients with *IDH*-mutant/*TERT*-wild-type tumors, and 2 of 5 patients with *IDH*-mutant/*TERT*-mutant tumors.

**Figure A8:**
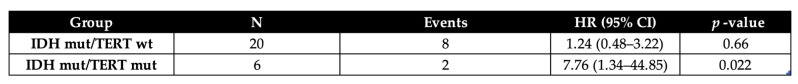
Survival outcomes according to combined IDH and TERT mutation status. Concurrent IDH/TERT mutations identified a high-risk subgroup with significantly worse overall survival (HR 7.76, 95% CI 1.34–44.85; *p* = 0.022).

Exploratory analyses were performed to evaluate the combined effect of *IDH* and *TERT* mutations. All *TERT* mutations occurred in chondrosarcomas harboring *IDH* mutations. While *IDH* mutation status alone was not associated with overall survival, the subgroup of patients (n = 6) with concurrent *IDH* and *TERT* mutations demonstrated significantly worse survival compared with other groups upon Cox regression (HR 7.76, 95% CI 1.34–44.85; *p* = 0.022) (Figure A8) and KM analysis (log-rank *p* = 0.03) (Figure 6). During follow-up, death occurred in 2 of 5 (40.0%) patients with concurrent *IDH/TERT* mutations compared with 8 of 20 (40.0%) patients with *IDH*-mutant/*TERT*-wild-type tumors and 9 of 27 (33.3%) patients with *IDH*-wild-type/*TERT*-wild-type tumors. These exploratory findings suggest that *TERT* mutations may identify a high-risk subset with *IDH*-mutant chondrosarcomas. Although an important observation, given the small number of co-mutated cases included in this analysis, these results should be interpreted cautiously and viewed as hypothesis-generating until validated in larger multi-institutional cohorts.

## 4. Discussion

In this study, we evaluated the genomic and clinicopathological determinants of survival in chondrosarcoma, with a primary focus on the prognostic role of *TERT* promoter mutations. Our findings suggest that *TERT* mutations are associated with aggressive tumor biology and may be linked to significantly decreased overall survival on exploratory univariable analysis. Notably, all *TERT*-mutated tumors in our cohort were grade 3, and the majority occurred in dedifferentiated chondrosarcoma, supporting *TERT* and its close relationship with tumor aggressiveness. These findings are consistent with prior evidence suggesting that *TERT* promoter mutations represent a late event in chondrosarcoma evolution. By enabling telomere maintenance, *TERT* mutations support sustained cellular proliferation, enabling tumor cells to bypass replicative senescence, and maintain long-term proliferative capacity. Our results therefore support a model in which *TERT* mutations arise in more advanced, biologically aggressive tumors, including dedifferentiated and high-grade lesions. The consistent co-occurrence of *TERT* and *IDH* mutations further suggests a biologically progressive model in which early *IDH*-driven tumorigenesis creates a permissive oncogenic environment that is followed by acquisition of *TERT* alterations that contribute to malignant progression and aggressive clinical behavior. Importantly, the molecular determinants of aggressive behavior in sarcoma likely extend beyond individual genomic alterations alone. Defects in antigen processing and presentation machinery differ substantially across sarcoma histotypes and may influence prognosis through mechanisms of immune escape [23]. These findings highlight the biological complexity underlying sarcoma progression and suggest that molecular biomarkers such as *TERT* alterations should be interpreted within the broader context of tumor evolution and host immune interactions.

Upon univariable analysis, *TERT* is significantly associated with worse survival. However, this effect was not maintained after adjusting for tumor size, grade, and subtype, suggesting that the prognostic impact of *TERT* may be mediated through its strong association with established markers of aggressive chondrosarcoma, rather than serving as an independent predictor. Importantly, these results do not necessarily diminish the clinical relevance of *TERT* but instead positions *TERT* as a biological marker that may reflect underlying tumor aggressiveness. This interpretation is consistent with recent studies from our institution demonstrating that biomarkers associated with aggressive tumor biology do not necessarily retain independent prognostic significance after multivariable adjustment for established clinicopathologic variables [24,25]. In contrast, *IDH* and *TP53* mutations did not demonstrate significant associations with overall survival in this cohort, despite being present across multiple histological subtypes. Although *IDH1* and *IDH2* mutations are recognized as early driver events in chondrosarcoma and share similar downstream effects through production of the oncometabolite D-2-hydroxyglutarate, whether *IDH* mutation subtype influences prognosis in chondrosarcoma remains incompletely understood. In exploratory sensitivity analyses, we did not identify significant differences in overall survival between *IDH1*- and *IDH2*-mutant tumors, although these analyses were limited by the small number of *IDH2*-mutant cases and wide confidence intervals. *IDH* alterations were also not significantly associated with recurrence outcomes. However, *TP53*-mutated tumors demonstrated a strong trend toward increased recurrence risk (OR = 7.00, *p* = 0.077), suggesting a potential relationship between *TP53*-associated pathway disruption and aggressive tumor behavior. Although not statistically significant, this finding may reflect the potential contribution of *TP53*-associated genomic instability to tumor progression and recurrence. Together, these findings emphasize the heterogeneity of genomic alterations in chondrosarcoma and suggest that not all mutations carry equivalent prognostic weight.

To confirm the internal validity of our cohort we analyzed the role of traditional clinicopathological factors in determining outcomes. Both dedifferentiated histology and higher tumor grade were strongly associated with decreased survival, consistent with prior literature. Additionally, advanced disease stage emerged as a critical determinant of prognosis, with stage 4 disease associated with markedly reduced survival compared to lower-stage tumors. Collectively, the alignment of these clinical variables with genomic findings reinforces a unified model in which tumor biology, histological phenotype, and disease burden influence patient survival outcomes.

Exploratory analysis was performed to signify the association of co-occurring mutations and survival outcomes. Particularly, we analyzed *IDH/TERT* co-mutations and found that all *TERT*-mutated tumors occurred within *IDH*-mutant cases. More importantly, this *IDH*-mutant/*TERT*-mutant subgroup demonstrated significantly worse survival within our cohort. Although limited by the small number of co-mutated tumors, these findings suggest that *TERT* alterations may identify a biologically aggressive subset within *IDH*-mutant chondrosarcoma. Rather than establishing definitive prognostic significance, these findings should be considered exploratory and hypothesis-generating until further investigation is validated in larger multicenter studies. Nonetheless, these findings align with emerging evidence supporting a multistep molecular progression model in chondrosarcoma, in which cumulative genomic alterations affecting telomere maintenance, epigenetic regulation, chromatin regulation, and cell cycle control contribute to aggressive transformation and metastatic potential. Moreover, our findings highlight the potential significance of integrating genomic profiling into risk stratification.

Although *TERT* did not retain independent prognostic significance after adjustment for established clinicopathologic variables, our findings suggest that *TERT* alterations may still provide clinically meaningful information regarding underlying tumor biology. In routine clinical practice, *TERT* testing could potentially serve as an adjunct to traditional prognostic factors such as tumor grade, histological subtype, and disease stage to help identify patients with biologically aggressive disease who may benefit from closer surveillance, multidisciplinary evaluation, or consideration for clinical trial enrollment [26,27,28]. When discussing genomic findings with patients, it is important to emphasize that *TERT* mutations should not be interpreted as deterministic predictors of outcome, but rather as molecular markers that may complement existing clinicopathologic features when estimating prognosis and guiding management decisions [29,30]. However, given the low prevalence of *TERT* mutations and the exploratory nature of the current evidence, routine implementation of *TERT* testing remains premature. Larger multicenter studies and external validation cohorts will be necessary to determine whether *TERT* testing provides incremental prognostic value beyond existing risk stratification models and should be incorporated into standard clinical practice.

It is important to note that the relatively small number of *TERT*-mutated cases and co-occurring *IDH*-mutant/*TERT*-mutant cases limited the statistical power of our study. Consequently, hazard ratio estimates should be interpreted cautiously, particularly for multivariable and subgroup analyses where the number of events was limited and confidence intervals were wide. Additionally, the retrospective single-center design and selective use of next-generation sequencing introduce the potential for selection bias and limit the generalizability of our findings. In clinical practice, NGS is more frequently performed in patients with advanced disease, dedifferentiated histology, atypical clinical behavior, or consideration for targeted therapies, potentially enriching our cohort for more biologically aggressive tumors and limiting the extent to which these findings can be extrapolated to the broader chondrosarcoma population. As a tertiary referral center, our institution may also disproportionately manage patients with advanced, recurrent, or anatomically complex tumors, further contributing to referral bias within the cohort. Survival outcomes were determined through retrospective review of the electronic medical record, and incomplete follow-up may have introduced additional selection bias if patients lost to follow-up differed systematically from those with available outcome data. Furthermore, incomplete genomic profiling for certain genes and the absence of an external validation cohort warrant cautious interpretation of these findings. Larger multicenter studies will be necessary to further validate these findings and better define the prognostic role of *TERT* alterations. Despite these limitations, the consistency of our findings with established clinicopathological variables supports the validity and potential clinical relevance of our results. Importantly, *TERT* promoter mutations were relatively uncommon within our cohort (7%), reflecting *TERT’s* low prevalence in chondrosarcoma rather than a sampling artifact.

## 5. Conclusions

*TERT* promoter mutations are associated with aggressive tumor biology and worse overall survival within this cohort, although their prognostic impact appears to be closely linked to established clinicopathologic factors such as tumor size, grade, and histological subtype. Notably, exploratory analyses demonstrated that concurrent *IDH* and *TERT* mutations were associated with worse survival outcomes and may identify a biologically aggressive subgroup of patients, suggesting that integrated molecular profiling may provide additional prognostic value beyond traditional clinicopathologic factors. These findings support a model in which *TERT* alterations may reflect biological mechanisms underlying tumor progression, dedifferentiation, and aggressive clinical behavior in chondrosarcoma. Given the limited number of *TERT*-mutated and *IDH/TERT* co-mutated tumors, larger multicenter studies are necessary to validate these exploratory findings and determine whether these alterations can be incorporated into future risk stratification models and therapeutic strategies.

## Figures and Tables

**Table 1:** Baseline demographic, clinicopathologic, and outcome characteristics of patients with clear cell, conventional, and dedifferentiated chondrosarcoma included in the study cohort. Values are presented as n (%) or mean ± standard deviation (SD). Continuous variables were calculated using available-case analysis.

Characteristic	Clear Cell (n = 3)	Conventional (n = 59)	Dedifferentiated (n = 29)	Overall (n = 91)	*p*-Value
**DEMOGRAPHICS**					
Age (years), mean ± SD	44.99 ± 11.79	55.94 ± 15.65	56.76 ± 12.07	55.76 ± 14.65	—
Male sex	2 (67%)	40 (68%)	9 (31%)	51 (56%)	—
Female sex	1 (33%)	19 (32%)	20 (69%)	40 (44%)	—
White race	2 (67%)	49 (83%)	28 (97%)	79 (87%)	—
Black race	1 (33%)	6 (10%)	1 (3.4%)	8 (8.8%)	—
Hispanic ethnicity	0 (0%)	21 (36%)	12 (41%)	33 (36%)	—
BMI (kg/m^2^), mean ± SD	27.91 ± 3.62	27.56 ± 5.48	28.11 ± 6.32	27.75 ± 5.67	—
**TUMOR CHARACTERISTICS**					
Appendicular location	2 (67%)	16 (27%)	17 (59%)	35 (38%)	—
Axial location	1 (33%)	43 (73%)	12 (41%)	56 (62%)	—
Tumor size (cm), mean ± SD	3.33 ± 0.58	7.41 ± 3.93	8.92 ± 3.46	7.72 ± 3.86	0.083
Localized disease	3 (100%)	57 (97%)	22 (76%)	82 (90%)	—
Metastatic at diagnosis	0 (0%)	2 (3.4%)	7 (24%)	9 (9.9%)	—
Positive surgical margins	0 (0%)	10 (17%)	2 (6.9%)	12 (13%)	0.492
Grade 2	2 (67%)	51 (86%)	7 (24%)	60 (66%)	<0.001
Grade 3	1 (33%)	8 (14%)	22 (76%)	31 (34%)	—
Final Stage I	0 (0%)	7 (12%)	0 (0%)	7 (7.7%)	0.2
Final Stage II	3 (100%)	23 (39%)	4 (14%)	30 (33%)	—
Final Stage III	0 (0%)	5 (8.5%)	3 (10%)	8 (8.8%)	—
Final Stage IV	0 (0%)	24 (41%)	22 (76%)	46 (51%)	—
**OUTCOMES**					
Recurrence after surgery	1 (33%)	32 (54%)	17 (59%)	50 (55%)	0.076
Local recurrence	1 (33%)	27 (46%)	14 (48%)	42 (46%)	—
Distant metastatic recurrence	0 (0%)	21 (36%)	15 (52%)	36 (40%)	0.105
Alive at final follow-up	3 (100%)	44 (75%)	16 (55%)	63 (69%)	—
Death at final follow-up	0 (0%)	15 (25%)	13 (45%)	28 (31%)	—
Disease-specific death	0 (0%)	14 (24%)	13 (45%)	27 (30%)	—
Loss to follow-up	0 (0%)	13 (22%)	9 (31%)	22 (24%)	—

**Table 2:** Distribution of genomic alterations across chondrosarcoma subtypes. Values are presented as n (%).

Characteristic	Clear Cell n = 3	Conventional n = 59	Dedifferentiated n = 29	Overall n = 91
**NGS**				
Yes	2 (67%)	32 (54%)	20 (69%)	54 (59%)
No	1 (33%)	27 (46%)	9 (31%)	37 (41%)
**IDH**				
Yes	0 (0%)	26 (44%)	21 (72%)	47 (52%)
No	3 (100%)	33 (56%)	8 (28%)	44 (48%)
**TP53**				
Yes	1 (33%)	6 (10%)	8 (28%)	15 (16%)
No	1 (33%)	26 (44%)	12 (41%)	39 (43%)
Not tested	1 (33%)	27 (46%)	9 (31%)	37 (41%)
**TERT**				
Yes	0 (0%)	1 (1.7%)	6 (21%)	7 (7.7%)
No	2 (67%)	31 (53%)	14 (48%)	47 (52%)
Not tested	1 (33%)	27 (46%)	9 (31%)	37 (41%)
**Epigenetic/Chromatin altered mutation**				
Yes	0 (0%)	8 (14%)	5 (17%)	13 (14%)
No	2 (67%)	24 (41%)	15 (52%)	41 (45%)
Not tested	1 (33%)	27 (46%)	9 (31%)	37 (41%)
**Cell cycle altered (CDKN2A, CDKN2B, RB1)**				
Yes	0 (0%)	3 (5.1%)	5 (17%)	8 (8.8%)
No	2 (67%)	29 (49%)	15 (52%)	46 (51%)
Not tested	1 (33%)	27 (46%)	9 (31%)	37 (41%)

## Data Availability

The data presented in this study are available upon reasonable request from the corresponding author. The data are not publicly available due to institutional privacy and ethical restrictions.

## Abbreviations

The following abbreviations are used in this manuscript:

TERT	Telomerase reverse transcriptase
IDH	Isocitrate dehydrogenase
NGS	Next-generation sequencing
HR	Hazards ratio
CDKN2	Cyclin-Dependent Kinase Inhibitor 2
RB1	Retinoblastoma 1
ATRX	Alpha Thalassemia/mental Retardation X-linked
KMT2	Lysine Methyltransferase 2
OR	Odds ratio
CI	Confidence interval
SD	Standard deviation
BMI	Body mass index
SETD2	SET domain-containing protein 2
BCOR	BCL6 corepressor gene
KM	Kaplan–Meier
TP53	Tumor Protein 53
